# High-dose methotrexate in ICU patients: a retrospective study

**DOI:** 10.1186/s13613-020-00693-5

**Published:** 2020-06-13

**Authors:** Sandrine Valade, Eric Mariotte, Elie Azoulay, Michael Darmon

**Affiliations:** 1 Medical ICU, APHP, Saint-Louis Hospital, Paris, France; 2 Université de Paris, Paris, France

**Keywords:** Methotrexate, Acute kidney injury, Outcome, Hematological malignancies, Drug-related side effects, Antineoplastic agents

## Abstract

**Background:**

High-dose methotrexate (HD-MTX) is commonly used in the treatment of solid tumors and hematological malignancies. Severe toxicities are frequent, leading to organ dysfunction and death. Risk–benefit ratio of using HD-MTX in critically ill patients is unknown. This study aims to describe MTX-induced toxicities and to assess outcome in ICU patients. We conducted a retrospective single-center study conducted in a university hospital ICU between January 2002 and December 2018. Consecutive patients treated by HD-MTX were included.

**Results:**

33 patients (24 men and 9 women) aged 48 years [34–63], were included. B cell lymphoma had been diagnosed in 31 patients (Burkitt, *n* = 14; diffuse large B-cell lymphoma with CNS (central nervous system) involvement, *n* = 9; primary CNS lymphoma, *n* = 5) and T-cell lymphoma in two patients. Patients were mainly admitted for coma (*n* = 14; 42%) or acute kidney injury (*n* = 8; 24%). MTX was administered at a median dose of 6.1 g [5–14]. Fourteen patients had concomitant medication interacting with MTX. Median MTX clearance was 4 days [4–5]. Frequent MTX-related complication were mucositis (*n* = 21, 64%), diarrhea (*n* = 14, 44%) or hepatic failure (*n* = 15, 45%). During ICU stay, 11 patients experienced acute kidney injury (KDIGO stage 3 [2–3]). Two patients received carboxypeptidase and three underwent dialysis. Overall, 19 patients (57%) required mechanical ventilation, 10 (30%) vasopressors. Hospital mortality was 30% (*n* = 10). Cox model identified MTX concentration 24 h after administration higher than 4.6 µmol/L as associated with hospital mortality (HR 6.7; 95% CI 1.6–27.3).

**Conclusions:**

To our knowledge, this is the first study assessing characteristics and outcome of critically ill patients receiving HD-MTX. MTX concentration at H24 was associated with hospital mortality. Despite underlying malignancy, ICU support of these patients was associated with a meaningful survival.

## Background

Intravenous high-dose methotrexate (HD-MTX) is commonly used in the treatment of hematological malignancies, particularly in high-grade lymphomas [1]. MTX-related toxicities are common, leading to organ dysfunction that can be very severe, and rarely to death [1]. Acute kidney injury (AKI) is frequently reported, affecting up to 35% of adult patients, mostly in relation with intratubular crystal formation or endothelial injuries [1, 2].

This drug requires a close monitoring and management of MTX-related toxicities relies mostly on preventing measures [1, 3]. AKI is frequent in critically ill patients with newly diagnosed high-grade hematological malignancies [4] and is associated with a high level of frailty. Whether ICU patients may be considered eligible for HD-MTX and risk–benefit ratio in this setting has never been assessed.

This primary objective of this study was to assess outcome in critically ill patients requiring HD-MTX infusion. Secondary objectives were to describe toxicities and risk factors of poor outcome in this setting.

## Methods

### Patients and data collection

We retrospectively reviewed the medical charts of all consecutive adult patients admitted to the intensive care unit of one university hospital from January, 1st, 2002 to December 31th, 2018, and who received HD-MTX for hematological malignancy or solid tumor. There were no exclusion criteria.

HD-MTX was defined by a single intravenous infusion greater than 500 mg/m^2^. The different stages of toxicity were defined according to the CTCAE [5]. MTX complete elimination was considered when MTX concentration was lower than 0.1 µmol/L.

This study was approved by a local ethic committee (Société de Réanimation de Langue Française, CE SRLF 19-01). According to French law, need for informed consent was waived.

### Statistical analysis

Results are described as medians and interquartile ranges (IQR) for quantitative variables and numbers and percentages for qualitative variables. We used a non-parametric Wilcoxon tests and Fisher exact tests for baseline univariate comparisons between two groups.

Cox regression model were performed to identify factors associated with hospital mortality. Variable selection was performed on a stepwise fashion, backward conditional model according to P value with entry *P* value of 0.2 and critical removal *P* value of 0.1. Proportional hazard assumption was checked in the final model.

All tests were two-sided, and *P* values less than 0.05 were considered significant. Analyses were done using R software version 4.3.4 (R Project for Statistical Computing, Wien, Austria) and with ‘Survival’ packages.

## Results

33 patients (24 men, 9 women) were included with a median age of 48 years [34–63]. All the patients had aggressive hematological malignancies and most of them (*n* = 31/33) had not received any antitumor treatment. B-cell lymphoma had been diagnosed in 31 patients (Burkitt [*n* = 14]; diffuse large B-cell lymphoma with CNS involvement [*n* = 9]; primary CNS lymphoma [*n* = 5]; primary effusion lymphoma [*n* = 2]; intravascular lymphoma [*n* = 1]) and T-cell lymphoma in two patients. Twelve (36%) had HIV infection, three had hypertension and one patient was diabetic (Table 1).

**Table 1:** Characteristics of patients at study inclusion

*N* (%) or median (IQR)	Survivors *n* = 23	Non survivors *n* = 10	*P* value
Demographics			
Age (years)	38 [31–51]	63.5 [51–69]	*0.013*
Male gender	14 (61%)	10 (100%)	0.058
Comorbidities			
HIV infection	9 (39%)	3 (12%)	0.914
Hypertension	2 (8.7%)	2 (20%)	0.74
Diabetes	1 (4.3%)	0	1
Hematological malignancy			0.52
B cell lymphoma	21 (91%)	10 (100%)	
Burkitt lymphoma	10	4	
Diffuse large B cell lymphoma	5	4	
Primary CNS lymphoma	4	1	
Primary effusion lymphoma	2	0	
Intravascular lymphoma	0	1	
T cell lymphoma	2 (8.7%)	0	
Reason for ICU admission			0.575
Neurological disorders	10 (43.5%)	4 (40%)	
Acute kidney injury	6 (26%)	2 (20%)	
Acute respiratory distress	3 (13%)	2 (20%)	
Cardiovascular failure	0	1 (10%)	
Other	4 (17%)	1 (10%)	
Biological tests			
Creatinine (µmol/L)	55 [39–72]	56 [47–86]	0.26
Albumin (g/L)	36 [31–38]	27.5 [24–31.8]	*0.045*
Bilirubin (µmol/L)	7.8 [6–13.2]	12 [6.9–13.4]	0.329
Leukocytes (G/L)	7.7 [5.1–8.9]	7.1 [1.5–10.0]	0.78
Hemoglobin (g/dL)	9.7 [8.3–12.5]	9.0 [8.1–10.3]	0.32
Platelets (G/L)	195 [150–255]	166 [111–272]	0.49
SOFA score	2 [1–5]	6 [4–9.5]	*0.022*
Treatments in the ICU			
Mechanical ventilation	10 (43.5%)	9 (90%)	*0.036*
Vasopressors	3 (13%)	7 (70%)	*0.004*
Renal replacement therapy	0	3 (30%)	*0.036*

*HIV* human immunodeficiency virus, *SOFA score* Sepsis-related Organ Failure Assessment, *ICU* intensive care unit

Patients were mainly admitted to the ICU for coma (*n* = 14; 42%) or acute kidney injury (*n* = 8; 24%). Five (15%) patients presented respiratory failure and only one patient had shock. Fifteen patients had a Glasgow Coma Scale of 12 or below and all except one presented with neurologic involvement related to lymphoma. SOFA score was 4 [1–5] at admission and 2 [1–5] the day of MTX infusion. All the patients except one presented a normal renal function at baseline (median serum creatinine = 55 µmol/L [41–74]), 12 experienced AKI episode in the last 3 months, including 8 requiring renal replacement therapy (RRT) (Table 1).

MTX was administered at a median dose of 3.4 g/m^2^ [2.6–7.4] and the median delay between ICU admission and MTX infusion was 4 days [2–7]. Twenty patients (61%) received concomitant intrathecal MTX. Other most commonly used chemotherapy drugs were cyclophosphamide (*n* = 23), doxorubicin (*n* = 23) and vincristine (*n* = 21). Median body mass index (BMI) was 24.9 [21.4–27.1] and median albumin level was 33 g/L [26–37]. Median creatinine level was 55 µmol/L [41–74] on the day of MTX administration. All patients received parenteral hyperhydration and alkalinization. Only 6 patients failed to achieve urine pH above 7.5 at least once within the first 24 h following MTX infusion. Folinic acid rescue was started 24 h after MTX infusion for all patients except one. The median time required until a complete elimination of MTX was 4 days [4–5]. Fourteen patients had concomitant medication interacting with MTX, mostly piperacillin–tazobactam (*n* = 8), proton-pump inhibitors (*n* = 9) or levetiracetam (*n* = 4). Seven patients presented serous effusions that required fluid removal (pleural effusions, *n* = 6 and ascites, *n* = 1).

More than 80% of patients (n = 27) experienced at least one MTX-related toxicity (Table 2). The most frequent MTX-related complications were mucositis (*n* = 21, 64%; median CTCAE grade 3 [2–4]), diarrhea (*n* = 14, 44%; median CTCAE grade 2 [2–3]) or liver tests disturbance (*n* = 15, 45%; median CTCAE grade 3 [2–4]). Following MTX infusion, the majority of patients developed neutropenia (*n* = 26) and acquired bacterial infections (*n* = 17, 51%). During ICU stay, one-third of patients (*n* = 11) experienced acute kidney injury (KDIGO stage 2.5 [2–3]) and median onset was reached 3.5 days [2–5] after MTX infusion. Eight patients also received concomitant nephrotoxic agents including contrast media (*n* = 3) and aminoglycosides (*n* = 3). Renal toxicity and MTX overdosage lead to carboxypeptidase G2 administration in 2 patients and need for RRT initiation in three.

**Table 2:** MTX-related toxicities and outcome

*N* (%) or median (IQR)	Survivors *n* = 23	Non survivors *n* = 10	*P* value
MTX infusion			
Median dose (g)	7.4 [4.9–14]	5.85 [5.1–10.5]	0.814
Time since ICU admission (days)	4 [1–7]	4 [2.2–8.7]	0.335
Interacting medications			
Mean number of medications (sd)	0.69 (0.89)	0.8 (0.92)	0.578
Piperacillin-tazobactam	4	4	
Proton-pump inhibitors	6	3	
Levetiracetam	4	0	
MTX-related complications	18 (78%)	9 (90%)	0.76
Including^a^			
Acute kidney injury	7 (30%)	4 (40%)	
Mucositis	15 (65%)	6 (60%)	
Diarrhea	6 (26%)	8 (80%)	
Liver tests disturbances	11 (48%)	4 (40%)	
MTX concentrations (µmol/L)			
MTX H24	3.1 [1.5–5.62]	19.5 [6.2–29.4]	0.052
MTX H36	5.4 [3–7.7]	16.7 [2.23–44.2]	0.643
MTX H48	0.4 [0.3–0.7]	1.6 [0.6–5.9]	0.103
MTX H72	0.3 [0.1–0.4]	0.6 [0.2–3.9]	0.143
MTX H96	0.07 [0.02–0.12]	0.34 [0.26–1.25]	0.013
Specific treatments			
Median dose of folinic acid rescue (mg)	200 [200–200]	320 [200–800]	*0.003*
Carboxypeptidase	1 (4.3%)	2 (20%)	0.436
Outcomes			
Length of ICU stay (days)	10 [6–18.5]	19.5 [10.7–25]	0.16
Length of hospital stay	64.5 [37–103]	36 [29–72.7]	0.13
End of life decision	2 (9%)	9 (90%)	< 0.001

*MTX* methotrexate, *ICU* intensive care unit

^a^Some patients experienced several MTX-related complications

Overall, 19 patients (57%) required mechanical ventilation, within a median time of 5 [1–12] days prior to MTX administration, and 10 (30%) vasopressors. Median length of ICU stay was 11 days [6–24]. Overall, ICU and hospital mortality were 18% (*n* = 6) and 30% (*n* = 10), respectively. Eighteen patients (55%) were alive 6 months after ICU discharge among whom, 15 (83%) had a complete and sustained hematological remission.

In univariate analysis, mortality was associated with older age (median age 63.5 years [51.25–68.75] vs 38 years [31–51.5, *p* = 0.013]), lower albumin level (27.5 g/L [24–31.75] vs 36 g/L [31–38], *p* = 0.045), and higher severity as assessed by SOFA score (6 [4.25–9] vs 2 [1–5], *p* = 0.022).

**Fig. 1:**
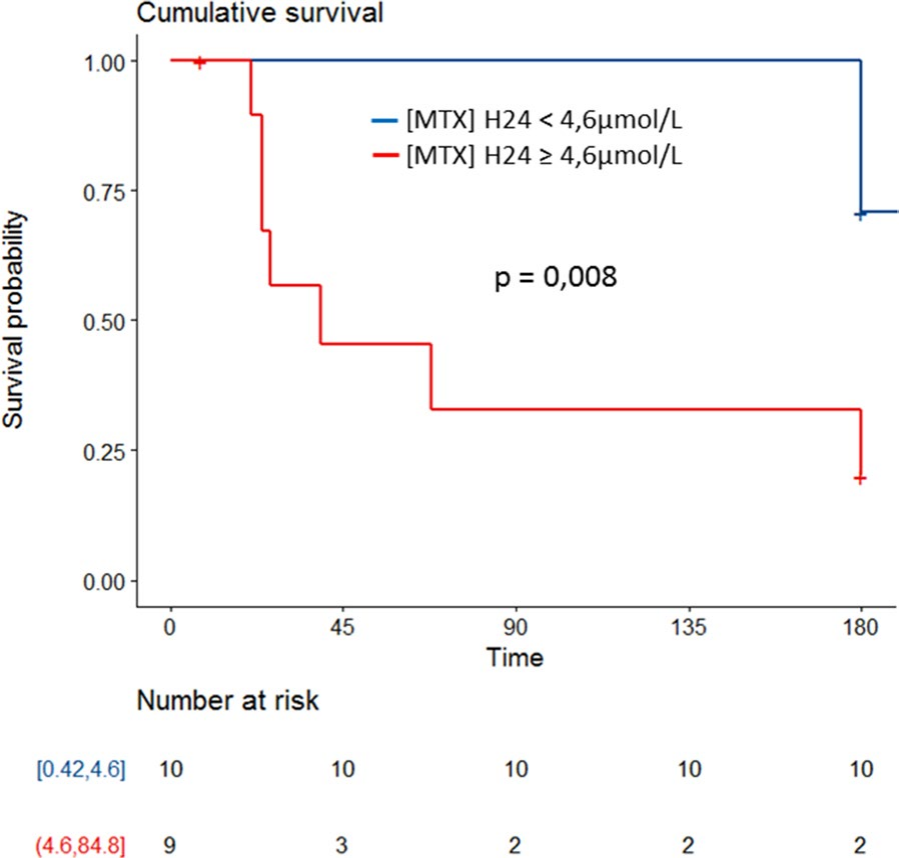
Adjusted influence of MTX dosage at H24

**Fig. 2:**
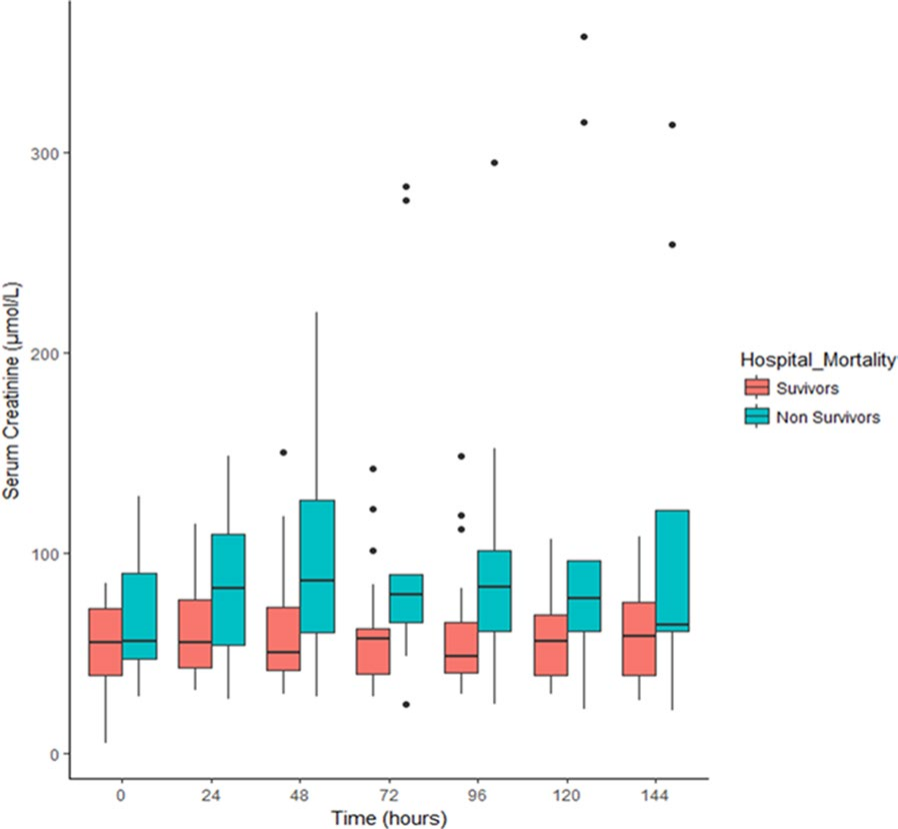
Relationship between creatinine and hospital mortality within the first week after MTX infusion

After adjustment for patients’ severity, MTX concentration 24 h after administration was independently associated with hospital mortality (HR if concentration above 4.6 μmol/L 6.7; 95% CI 1.6–27.3) (Table 3) (Fig. 1). In non-survivors, creatinine levels were significantly higher the day after MTX administration (*p* = 0.0017) and during the first week (*p* = 0.026) (Fig. 2).

**Table 3:** Variables associated with hospital mortality after adjustment

Variables	Hazard ratio	95% confidence interval	*P* value
MTX at H24 (4.6–84.8)	6.7	(1.62–27.3)	0.008
SOFA score	1.07	(0.89–1.27)	0.47

## Discussion

To our knowledge, this is the first study assessing benefits and risk of HD-MTX in critically ill patients. This study underlines the high rate of risk factors for HD-MTX toxicity and the high rate of MTX-related toxicities. Our results also underline that 6-month survival may be obtained in 55% of the patients and that complete remission may be obtained in 83% of them.

In the literature, classically 2 to 12% of non-ICU patients are reported to develop renal failure following HD-MTX. In fact up to 35% of patients experienced AKI, with a large heterogeneity according to the studied population, HD-MTX protocols and AKI criteria [6]. In critically ill patients, AKI incidence also varies widely from 22 to 67%, discrepancies mainly relying on the definition applied [7]. In our study, we found a high rate of AKI as one in three patients experienced renal failure. This is in line with a previous study in which the authors showed that two-thirds of critically ill patients with newly diagnosed aggressive hematological malignancies developed AKI [4]. Our results highlight that MTX-induced renal toxicity is very frequent in ICU patients.

Survival of patients with hematological malignancies has improved over the past decades and an increasing number of patients may need ICU admission [8]. In previous studies in cancer patients receiving chemotherapy in the intensive care unit, hospital mortality is reported around 40% [9]. Our study, concurrently suggests the feasibility of HD-MTX in this setting, demonstrating that despite the high toxicity rate, a 6-month survival rate of 55% may be achieved, the majority of survivors achieving complete remission.

Dose–toxicity relationship of MTX has been descried previously. The most commonly used threshold is a concentration greater than 10 µmol/L 24 h after MTX infusion or greater than 1 µmol/L at H48 [3]. Evans et al. [10] previously demonstrated that values above 10 µmol/L 24 h after the start of MTX infusion were associated with an increased risk of toxicity. As no published data exist in ICU patients, optimal early MTX concentrations predicting the outcome remain unknown.

This study suffers however several limitations. First, due to its retrospective design, exhaustivity of data was limited. Thus, exact assessment of optimal MTX concentration predicting poor outcome could not be assessed. Second, the small sample size led to limited statistical power, negative findings needing to be interpreted cautiously. Moreover, patients deemed eligible to HD-MTX infusion were likely to be selected according to performance status and clinical severity. Despite these limits, our study demonstrates feasibility of HD-MTX with meaningful chances of long-term survival and complete remission.

## Conclusion

This study demonstrates feasibility of HD-MTX in a selected group of critically ill cancer patients. Although the toxicity rate was high, long-term survival was achieved in more than half of the patients and complete remission was achieved in most of these later. Additional studies are needed to allow better identification of patients at high risk of toxicity.

## Supplementary information

**Additional file 1.** Additional materials.

**Additional file 2: Table S1.** Standard folinic acid rescue protocol (adapted from T Balloy et al. Modalités de prise en charge des intoxications aiguës par le méthotrexate haute dose. Journal de Pharmacie Clinique. 2007;26(4):253–260. doi:10.1684/jpc.2007.0070).

**Additional file 3: Table S2.** Criteria for carboxypeptidase use according to the French National Agency for Medicines and Health Products Safety.

## Data Availability

The dataset supporting the conclusions of this article is included within the article (and its Additional files 1, 2, and 3).

## Abbreviations

AKI: Acute kidney injury
BMI: Body mass index
CNS: Central nervous system
CTCAE: Common Terminology Criteria for Adverse Events
HD-MTX: High-dose methotrexate
ICU: Intensive care unit
MTX: Methotrexate
SOFA: Score, sepsis-related organ failure assessment
