# Supplementary material for: High-dose methotrexate in ICU patients: a retrospective study
Source: Ann Intensive Care. 2020 Jun 13;10:81. doi: 10.1186/s13613-020-00693-5 (PMC7293713; doi:10.1186/s13613-020-00693-5)
Supplement: Supplementary file 1 — Additional file 1. Additional materials. [file 13613_2020_693_MOESM1_ESM.docx]

**ADDITIONAL MATERIAL**

**Additional data on methodology**

Data reported in tables and figures were abstracted from the medical records. Clinical and laboratory data at ICU admission were collected, as well as organ failure and specific management during ICU stay. We also collected data on MTX administration, potential interacting medications and kidney function during the first week after MTX infusion. Standard folinic acid rescue protocol was started 24 hours after MTX administration (Table S1). Carboxypeptidase was available in France from 2007 under temporary authorization for use. Criteria of carboxypeptidase use were based on the French National Agency for Medicines and Health Products Safety (ANSM) recommendations (Table S2) and medical consensus. We recorded the treatments used to manage MTX-related toxicities: aminoglycosides, contrast media, colimycin and high-dose acyclovir were evaluated as nephrotoxic agents, whereas piperacillin-tazobactam, proton-pump inhibitors and levetiracetam were considered as concomitant drugs interacting with MTX elimination. SOFA score was applied to assess severity (JL Vincent et al. The SOFA (Sepsis-related Organ Failure Assessment) score to describe organ Dysfunction / failure. Intensive Care Med 1996;22:707-710). ICU and hospital mortality were available for all patients, the duration of follow-up was 6 months.

**Additional data on statistical analysis**

Survival curves were constructed according to the Kaplan–Meier method. Comparison according to timing of admission was performed using the log-rank test.

Changes in serum creatinine and in MTX serum concentration were assessed using ANOVA trend test.
