# Supplementary material for: High-dose methotrexate in ICU patients: a retrospective study
Source: Ann Intensive Care. 2020 Jun 13;10:81. doi: 10.1186/s13613-020-00693-5 (PMC7293713; doi:10.1186/s13613-020-00693-5)
Supplement: Supplementary file 2 — Additional file 2: Table S1. Standard folinic acid rescue protocol (adapted from T Balloy et al. Modalités de prise en charge des intoxications aiguës par le méthotrexate haute dose. Journal de Pharmacie Clinique. 2007;26(4):253–260. doi:10.1684/jpc.2007.0070). [file 13613_2020_693_MOESM2_ESM.docx]

**Table S1: Standard folinic acid rescue protocol (adapted from T Balloy et al. Modalités de prise en charge des intoxications aiguës par le méthotrexate haute dose. Journal de Pharmacie Clinique. 2007;26(4):253-260. doi:10.1684/jpc.2007.0070)**

| **Time since MTX administration** | **MTX dosage (µmol/L)** | **Daily folinic acid dosage regimen (mg/day)** |
| --- | --- | --- |
| 24 hours  48 hours  72 hours  > 72 hours | 0.05-10  > 10  0.05-1  1-5  5-10  >10  0.05-0.5  0.5-1  1-5  >5  0.05-0.1  0.1-0.5  0.5-1  >1 | 100  200  100  200  400  800  100  200  400  800  100  200  400  800 |
