# Supplementary material for: High-dose methotrexate in ICU patients: a retrospective study
Source: Ann Intensive Care. 2020 Jun 13;10:81. doi: 10.1186/s13613-020-00693-5 (PMC7293713; doi:10.1186/s13613-020-00693-5)
Supplement: Supplementary file 3 — Additional file 3: Table S2. Criteria for carboxypeptidase use according to the French National Agency for Medicines and Health Products Safety. [file 13613_2020_693_MOESM3_ESM.docx]

**Table S2: criteria for carboxypeptidase use according to the French National Agency for Medicines and Health Products Safety**

| **Patients who have received high-dose Methotrexate in the management of hematological malignancies** |
| --- |
| Methotrexate concentration greater than 10 μmol/L at least 42 hours after Methotrexate administration  OR  Methotrexate concentration greater than 2 standard deviations of the mean dose-specific elimination curve of Methotrexate, at least 12 hours after Methotrexate administration  AND  Creatinine level > 1.5 times baseline or glomerular filtration rate < 60ml/min |
